# The Impotence of Non-Brownian Particles on the Gel Transition of Colloidal Suspensions

**DOI:** 10.3390/polym9090461

**Published:** 2017-09-19

**Authors:** Samantha L. Morelly, Maureen H. Tang, Nicolas J. Alvarez

**Affiliations:** Department of Chemical and Biological Engineering, Drexel University, Philadelphia, PA 19104, USA; slm394@drexel.edu (S.L.M.); mhtang@drexel.edu (M.H.T.)

**Keywords:** rheology, colloidal suspension, colloidal gel, battery manufacturing, battery rheology

## Abstract

The ability to predict transitions in the microstructure of mixed colloidal suspensions is of extreme interest and importance. The data presented here is specific to the case of battery electrode slurries whereby the carbon additive is reported to form strong colloidal gels. Using rheology, we have determined the effect of mixed particle systems on the critical gel transition $\phi_{\mathrm{gel}}$. More specifically, we show that the introduction of a high volume fraction of large non-Brownian particles has little to no effect on $\phi_{\mathrm{gel}}$. Although $\phi_{\mathrm{gel}}$ is unchanged, the larger particles do change the shape of the linear viscoelasticity and the nonlinear yielding behavior. There are interesting similarities to the nonlinear behavior of the colloidal gels with trends observed for colloidal glasses. A comparison of experimental data and the prediction from theory shows that the equation presented by Poon et al. is able to quantitatively predict the transition from a fluid state to a gel state.

## 1. Introduction

Coatings of particle–polymer composites are ubiquitous in a myriad of applications, including lithium-ion and other advanced battery technologies that are predicted to play substantial roles in growing concerns over the energy and environmental landscape [1]. Both material- and device-level limitations play a role in determining battery performance, lifetime, and cost. One of the greatest of these limitations is charge transport. Electrodes must have sufficiently fast electron and ion transport to utilize the electrochemically active material and prevent resistive losses. The rate of transport is determined not only by material properties, but also by the electrode microstructure [2,3]. Recent studies suggest that the final “dry” microstructure is determined by the initial “wet” microstructure of the colloidal slurries formed during electrode processing [4,5,6,7,8,9,10,11,12,13,14,15,16,17].

Considerable effort has been dedicated to the effects of electrode slurry composition and formulation on battery performance. In order to eliminate transport effects when developing new materials, academic battery formulations can include up to 20 wt % conductive additive [18]. Industrial formulations have much lower additive concentrations, typically less than 5 wt %, to minimize the mass and volume of electrochemically inert components. For these conductive additive-starved systems, achieving the optimal electrode microstructure is much more critical. Research has shown that, when colloidal electrode slurries form gel-like microstructures, the resulting batteries perform superior to those formed from fluid-like microstructures [4,5,9]. In gel-like slurries, the formation of a percolating network of conductive additive provides pathways for rapid electronic transport and prevents polymer migration during drying [19,20,21]. Inducing gelation in electrode slurries is therefore paramount in manufacturing high-performing batteries.

Wet battery slurries are known to form colloidal gels induced by polymer depletion interactions. Electrode slurries include polymer binder to improve the mechanical strength of the electrode and the adhesion between dried electrode and current collector (e.g., aluminum foil) [22]. This polymer has the added effect of inducing an interparticle attraction potential, *U*_dep_, which promotes particle aggregation [23,24,25,26,27,28,29,30,31,32,33]. The strength of the interparticle interaction scales strongly with polymer concentration. In the limit of a high polymer concentration, *U*_dep_ >> *k*_B_*T* and the particles form permanent linkages. Battery slurries are expected to be in this limit. Typical polymer concentrations in battery slurries reach up to 1–2 wt % in industrial formulations and up to 15 wt % in academic formulations [18].

For the case of high polymer concentration, *U*_dep_ >> *k*_B_*T*, Poon et al. derived an analytical expression for the critical gelation volume fraction, $\phi_{\mathrm{gel}}$, taking into account gravity and is given by

(1) $$\phi_{\mathrm{gel}}={\left(\frac{9k_{b}T}{2\pi \;\Delta \rho ga^{4}}\right)}^{\frac{D_{f}-3}{D_{f}+1}}\;$$

where *a* is the particle radius, $D_{f}$ is the fractal dimension, and Δρ is the difference between solvent and particle density [30,32]. Equation (1) is derived by relating the gravitational Peclet number, *P*e_g_, to the critical gelation cluster radius, $R_{\mathrm{gel}}$. In other words, Equation (1) determines the minimum volume fraction at which volume spanning aggregation occurs before particles settle due to gravity. In the case of battery slurries, nanometer-scale particles of conductive carbon black (CB, ρ = 1.9 g/cm^3^) are mixed with micrometer-scale particles of mixed-metal oxides such as Ni_0.33_Mn_0.33_Co_0.33_O_2_ (NMC, ρ = 5.18 g/cm^3^). Due to the high densities of CB and NMC, we expect a competition between aggregation kinetics and gravitational settling of particles. Active material particles typically range from 1 to 50 µm in diameter and by definition are non-Brownian due to size and density, i.e., *P*e_g_ > 10. Note that Equation (1) predicts that there is no critical gel concentration for active material due to the dominant gravitational force. Conductive additive particles rarely exceed 100 nm in diameter and are typically considered colloidal. The smaller CB particles could form a gel depending on their size and aggregation kinetics. It is not clear from current colloidal theory whether the mixture of CB and NMC particles should form a volume spanning gel, but experimental evidence suggests they do [4,9]. We hypothesize that, when such a particle mixture forms a gel, it must be primarily due to a critical volume fraction of small conductive additive particles.

Currently, colloidal gel theory does not have a prediction for the critical volume fraction of a binary population of particle sizes. Experimental work has focused on the study of bimodal particle size distributions and their effect on aggregation behavior. Reported findings include a measure of dynamic viscosity as a function of particle size distribution [33,34,35,36]. There is little experimental work on the effects of particle size distributions on the fundamental microstructure of the underlying mixture. Additionally, these previous studies have considered only neutrally buoyant particles of the same chemistry, while many applications, including battery slurries, contain dissimilar non-buoyant particles. The lack of knowledge predicting fluid microstructure for colloidal slurries with multiple particle populations limits the ability of designing optimal processes for electrode manufacturing. In this work, we present a fundamental study of gelation in polydisperse systems. We determine $\phi_{\mathrm{gel}}$ experimentally for a system of nanometer-scale colloidal carbon black (CB), a common conductive additive in lithium-ion batteries, and for a mixed system of CB and micrometer-scale non-Brownian Ni_0.33_Mn_0.33_Co_0.33_O_2_ (NMC), a state-of-the-art battery material. We find that non-Brownian particles do not participate in the percolating network and therefore leave the value of $\phi_{\mathrm{gel}}$ relatively unchanged.

## 2. Materials and Methods

Materials and Sample Preparation: Nano-sized carbon black, CB, was used as received (Super C65, Timcal, Bodio, Switzerland). The reported particle size was 100 nm [37]. Lithium Nickel Manganese Cobalt Oxide (Ni_0.33_Mn_0.33_Co_0.33_O_2_), NMC, was used as received (NM-3100, Toda America, Battle Creek, MI, USA). The reported average particle size was 10 microns. Polyvinylidene difluoride, PVDF, *M*_w_ = 380,000 (Arkema, Kynar 301F, King of Prussia, PA, USA) was used as received. 1-Methyl-2-pyrrolidinone, NMP, was used as the solvent (Sigma Aldrich, purity ≥99.0%, St. Louis, MO, USA). Sodium dodecyl sulfate, SDS, was used as received (Alfa Aesar, Tewksbury, MA, USA). The polymer concentration, *c*_p_ = 48 mg/mL for all experiments and the volume fraction of carbon black varied with respect to solvent. In the mixed particle case, the volume fraction of NMC was maintained at 0.26 with respect to solvent only. $\phi_{\mathrm{NMC}}=0.26$ represents a tradeoff between an appropriate viscosity for coating electrodes and limited solvent for faster electrode drying [18,38]. Samples were mixed in a planetary mixer (Thinky Corporation, ARE-250, Laguna Hills, CA, USA). The mixing protocol is as follows: (1) The binder and solvent were mixed at 1800 rpm for 10 min and (2) CB and NMC were added separately to the polymer solution and mixed at 1800 rpm for 7.5 min.

Rheological Characterization: Oscillatory rheometry is performed on an AR-2000 rheometer (TA Instruments, Newcastle, DE, USA) using the Peltier plate setup and a 40 mm parallel plate geometry at *T* = 25 °C. Samples were loaded onto the parallel plate either by pouring or gently with a spatula. The geometry was lowered slowly to ensure no entrainment of air bubbles. The parallel plate geometry was chosen to minimize confinement effects. The linear viscoelastic measurements were measured, as were various gap heights ranging from *h* = 300 μm to 1 mm in order to determine gap effects. The results show that, at *h* ≤ 500 μm, gap effects are absent. All reported measurements were independently confirmed using the ARES G2 rheometer (TA Instruments, Newcastle, DE, USA) with a 25 mm parallel plate geometry. The data reported only reflect measurements made with the AR-2000, (TA Instruments, Newcastle, DE, USA). Oscillatory strain sweep (fixed angular frequency, ω = 1 rad/s) and frequency sweep (fixed strain amplitude, γ = 0.003) measurements were performed after a waiting time of 5–10 min to ensure sample equilibration or longer to ensure the normal force returned to zero.

Although the time scale of gelation was not the main focus of this paper, time sweeps were performed on samples with ϕ_CB_ = 0.025, ϕ_NMC_ = 0, and ϕ_CB_ = 0.025, ϕ_NMC_ = 0.26. Samples were loaded onto the AR-2000 (TA Instruments, Newcastle, DE, USA) gently with a spatula and placed on a Peltier plate (TA Instruments, Newcastle, DE, USA). Samples were tested at 25 °C with a 40 mm diameter parallel plate geometry at a gap height of 500 μm. The samples were pre-sheared at a rate of 100 rad/s for 5 min prior to measuring the time sweep recovery. Due to the length of the experiments a silicone oil bath was used to prevent solvent evaporation. *G*’ and *G*” were reported at 1 rad/s and 0.3% strain over a 3 h period, after the samples were sheared at 100 1/s for 5 min.

Particle Size Characterization: DLS and TEM samples of CB in water were prepared by mixing 0.04 mg of CB in 20 mL of a 0.17 mM SDS solution. Measurements were made below the critical micelle concentration of SDS (CMC = 8.2 mM). The SDS was used to stabilize the CB suspension; the particles aggregate and settle out otherwise. DLS measurements were made on a Brookhaven D90, (Brookhaven Instruments, Holtsville, NY, USA) using a 90° scattering angle. Samples were sonicated for 5 min before DLS measurements were taken in an attempt to break up secondary aggregates. The number average particle diameter was determined as the mean of the best fit distribution, *d*_CB_ = 100 nm. TEM pictures were carried out on a JEOL JEM2100 (Peabody, MA, USA). Samples were prepared by dip drying of CB SDS solutions onto a TEM grid (Pacific Grid Tech, 400-mesh, San Francisco, CA, USA). The NMC particles were imaged using a Zeiss Supra 50 VP Scanning Electron Microscope (Zeiss Group, Pleasonton, CA, USA) (SEM).

Microstructure Characterization: Samples were deposited on a glass slide and gently sandwiched with another glass slide for optical imaging. Images were collected using a stereo microscope with digital camera, model MU130 (AMSCOPE, Irvine, CA, USA) with 1× and 4× magnification in transmission mode. The images were analyzed using Matlab^®^ (Mathworks, Natick, MA, USA) and the “boxcount” package to determine the fractal dimension.

## 3. Results

### 3.1. Direct Observations

**Control Sample:** A $\phi_{\mathrm{CB}}$ = 0.005 NMP solution in the absence of PVDF was observed as a control. After mixing for 7.5 min, the system was an opaque black liquid. For the first 2 h, there was no noticeable change in fluid characteristics. After 3 h, the sample exhibited noticeable sedimentation when light was shown through the sample.

**CB Only***:* Mixtures of CB and PVDF in NMP were observed for 0.004 $\leq \;\phi_{\mathrm{CB}}\leq$ 0.009. In all cases, the samples were opaque black and indistinguishable from the control sample described above. There was no noticeable change in the sample characteristics within the first 2 h of observation. After 24 h, a translucent top layer and a slight sedimentation bottom layer for samples 0.004 $\leq \phi_{\mathrm{CB}}\leq$ 0.007 appeared. The sedimentation layer was significantly subtler than observed for the control. For samples $\phi_{\mathrm{CB}}>0.009$, no noticeable change in sample characteristics were observed over a 48 h observation period. During the loading of samples onto the rheometer, there was a decrease in the ease of pouring for samples with $\phi_{\mathrm{CB}}\geq$ 0.008. Samples with $\phi_{\mathrm{CB}}\geq$ 0.02 required a spatula to be loaded onto the rheometer.

**Mixed Particles:** Mixtures of CB, NMC ($\phi_{\mathrm{NMC}}=0.26)$, and PVDF in NMP were observed for $\phi_{\mathrm{CB}}=\;$0.008, 0.01, 0.012, 0.013, 0.014, 0.015, and 0.02. In all cases, the samples were opaque black and indistinguishable from the above-mentioned cases. After 30 min, sedimentation was observable for $\phi_{\mathrm{CB}}$ = 0.008. After 1 h, sedimentation was visible for 0.008$\;<\phi_{\mathrm{CB}}\leq \;$0.01. After 24 h, sedimentation was observable in samples up to $\phi_{\mathrm{CB}}=$ 0.015. At $\phi_{\mathrm{CB}}=0.013$, an evident increase in viscosity was observed. Overall, the sedimentation layer thickness appeared to decrease with increasing $\phi_{\mathrm{CB}}$. After the initial observed changes, there were no additional changes observed up to 48 h. Similar to the CB-only case, when $\phi_{\mathrm{CB}}\geq$ 0.02, the suspension showed no sedimentation and did not flow with gravity; i.e., a spatula was required to load the samples onto the rheometer.

### 3.2. Material Characterization

**Figure 1:**
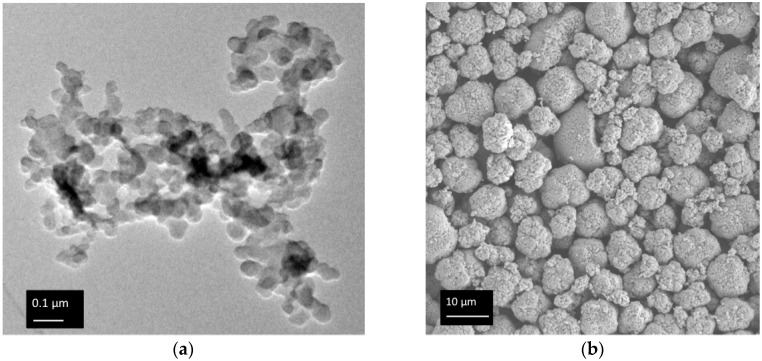
(**a**) TEM of a carbon black nanoparticle aggregate showing primary particle diameters ranging from 30 to 100 nm; (**b**) SEM of NMC microparticles showing an average particle size of 10 µm.

A representative TEM image of solution-dried CB nanoparticles forming an aggregate structure is shown in Figure 1a. The primary CB particles have diameters ranging from 15 to 200 nm, as probed using ImageJ software (NIH, Bethesda, MD, USA). This is in good agreement with the number previously reported in the literature [37]. The NMC particle diameter was estimated from SEM (Figure 1b) using ImageJ software to be approximately 10 µm. The SEM image and size sampling is in good agreement with the manufacturer’s specifications of 10 µm. Although some NMC particles in Figure 1b were observed to be only 2–3 µm in diameter, these particles are still considered non-Brownian because the high density of NMC results in *P*e_g_ > 10 for particles larger than 550 nm.

### 3.3. Rheology at Fixed c_p_

**Figure 2:**
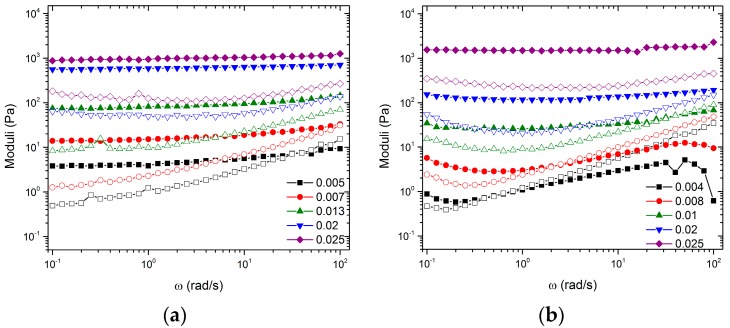
Frequency dependence of *G*’ (filled symbols) and *G*’’ (open symbols) as a function of $\phi_{\mathrm{CB}}$ for (**a**) $\phi_{\mathrm{NMC}}=0$ and (**b**) $\phi_{\mathrm{NMC}}=0.26$.

The linear viscoelastic (LVE) responses of samples with increasing $\phi_{\mathrm{CB}}$ and $\phi_{\mathrm{NMC}}=0$ are shown in Figure 2a. For ω < 20 rad/s, $\phi_{\mathrm{CB}}$ = 0.005 shows *G*’ > *G*’’ and a slight dependence of moduli on frequency. At ω = 20 rad/s, there is a crossover in modulus, at which point *G*’’ > *G*’ for larger frequencies. This overall LVE response is indicative of a weak gel. For increasing $\phi_{\mathrm{CB}}$, the LVE increases in magnitude and the crossover shifts to higher frequencies. For $\phi_{\mathrm{CB}}>0.007$, there is a significant jump in the magnitude of *G*’ and a shift in the crossover to higher frequencies (outside of the measured window). For $\phi_{\mathrm{CB}}$ > 0.007, there is a steady increase in the modulus with $\phi_{\mathrm{CB}}$, and *G*’’ begins to show a decreased dependence on frequency in the measured window, indicative of a strong gel.

Figure 2b shows the LVE response of samples with increasing $\phi_{\mathrm{CB}}$ and $\phi_{\mathrm{NMC}}=0.26$. Samples with $\phi_{\mathrm{CB}}<0.01$ show significant settling after 12 h. For these samples, the reported data represent the instantaneous response immediately after mixing. Samples $\phi_{\mathrm{CB}}$ = 0.004 and 0.008 exhibit a dependence of *G*’ on frequency and a crossover at ω = 0.03 and 3 rad/s, respectively. $\phi_{\mathrm{CB}}>0.008$ show a significant increase in modulus and an invariance of *G*’. For $\phi_{\mathrm{CB}}=0.01,$ *G*’ and *G*’’ cross at ω = 40 rad/s. For $\phi_{\mathrm{CB}}>0.01$, a crossover of *G*’ and *G*’’ is not observed in the measured frequency window.

**Figure 3:**
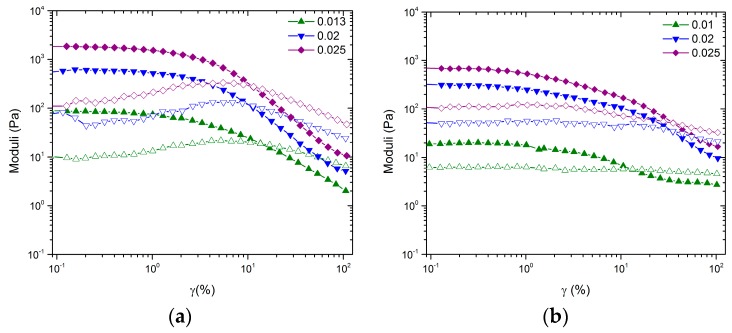
Amplitude dependence of *G*’ (filled symbols) and *G*’’ (open symbols) as a function of $\phi_{\mathrm{CB}}$ at 1 rad/s for (**a**) $\phi_{\mathrm{NMC}}=0$ and (**b**) $\phi_{\mathrm{NMC}}=0.26$.

Figure 3a shows the amplitude sweep for a fixed frequency, ω = 1 rad/s, and select $\phi_{\mathrm{CB}}$ samples are presented in Figure 2a. While all samples exhibit a linear response at low amplitudes, a clear crossover is observed for all three samples at γ=10%. The crossover amplitude indicates the point of gel breakup. The observed maximum of *G*’’ at the crossover amplitude is indicative of a hard sphere colloidal glass response. Figure 3b shows the amplitude sweep for a fixed frequency, ω = 1 rad/s, for select $\phi_{\mathrm{CB}}$ samples presented in Figure 2b. The concentrations shown in Figure 3b show a small linear viscoelastic window followed by a shallow decrease of *G*’ with amplitude. *G*’’ shoes no dependence on amplitude where γ<3% and a decreasing trend at high amplitude. *G*’’ does not appear to go through a maximum, but rather begins to decrease at γ=3% until a crossover is observed between γ=10% and 30% depending on the value of $\phi_{\mathrm{CB}}$.

**Figure 4:**
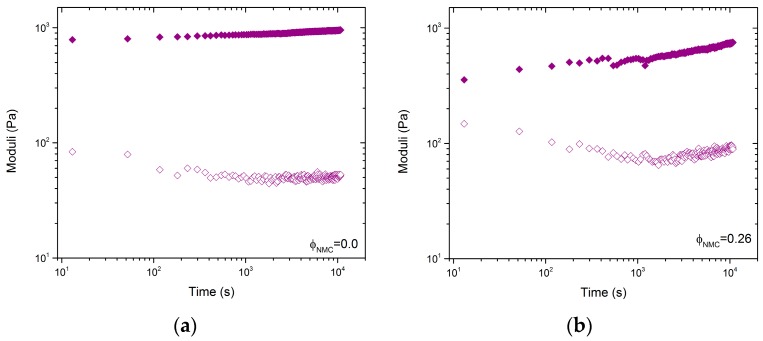
Time sweeps of samples with $\phi_{\mathrm{CB}}=0.025$ recovery over 3 h after 5 min of shearing at 100 1/s. *G*’ is represented by filled symbols and *G*” by hollow symbols. (**a**) Sample with $\phi_{\mathrm{NMC}}=0$; (**b**) Sample with $\phi_{\mathrm{NMC}}=0.26$.

Figure 4a,b shows recovery time sweeps for samples with a fixed CB concentration of 0.025, and $\;\phi_{\mathrm{NMC}}=$ 0 and 0.26, respectively. Figure 3a,b show that shearing at 100 1/s should be sufficient to break up the aggregate structures. However, the moduli show almost immediate recovery after cessation of the shear. This is typical of strongly adsorbed aggregates formed via irreversible DLCA. There does appear to be a slight recovery of the mixed particle system that is not as prevalent in the CB-only samples, which suggests that the relaxation time of the mixed system is larger than the CB-only system. The detailed mechanistic analysis performed below will shed light on these observations. Note that, since settling is observed in samples for $\;\phi_{\mathrm{CB}}<0.02$, time sweep data were not measured.

## 4. Discussion

Zaccone et al. suggest that the mechanism of aggregation can be readily determined via analysis of the relaxation spectrum *H*(τ) as a function of time [39]. There are three unique aggregation mechanisms: irreversible, reversible, and chemical aggregation. The slope of *H*(τ) at low frequency determines which mechanism is dominating. A slope *n* < 0 indicates reversible aggregation, *n* = 0 indicates irreversible aggregation, and *n* > 0 indicates chemical aggregation. To turn *G*’ and *G*’’ data into *H*(τ), a discrete relaxation spectrum, *G*(τ), was fit to the frequency sweep data reported in Figure 2. The Maxwell modes were then used to determine *H*(τ) [40]:

$$H\left(\tau \right)=\overset{N}{\underset{i=0}{\sum }}g_{i}\delta \left(1-\frac{\tau }{\tau_{i}}\right).$$

**Figure 5:**
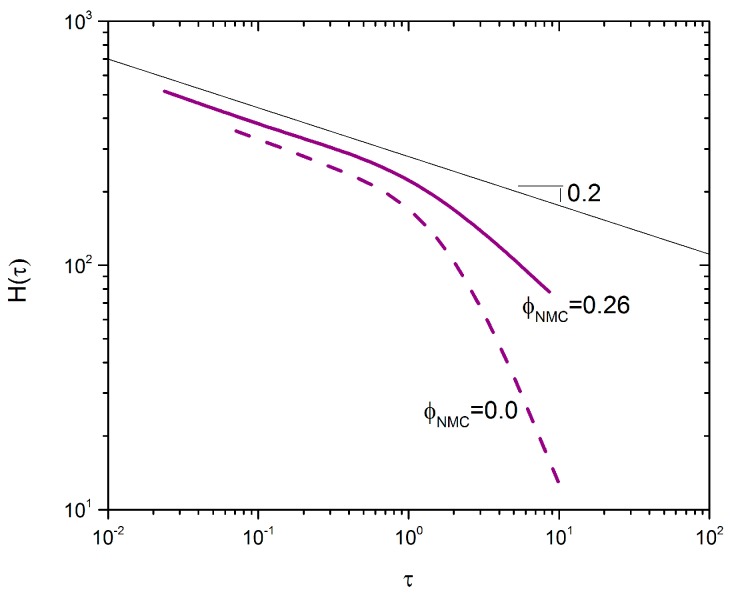
Relaxation time spectra for $\phi_{\mathrm{CB}}=0.025$ with $\phi_{\mathrm{NMC}}=0.26$ and $\phi_{\mathrm{NMC}}=0,$ represented by a solid and dashed line, respectively. The black line is a representative power law line with a slope of 0.2.

Figure 5 shows *H*(τ) for samples with $\phi_{\mathrm{CB}}=0.025,\;\phi_{\mathrm{NMC}}=0.26\;\mathrm{and}\;\phi_{\mathrm{CB}}=0.025,\;\phi_{\mathrm{NMC}}=0$. The two curves with and without NMC particles have very similar negative slopes at low frequency, *n* = –0.2, which suggests a mixed aggregation mechanism that is closer to irreversible aggregation [39]. This is in-line with the time sweep data in Figure 4, which shows very fast recovery of the modulus—typical of irreversible DLCA [39]. Furthermore, the inflection point, which indicates the maximum relaxation time scale, $\tau_{c}\sim 1\;s$, is of the same order of magnitude in both cases. This suggests that the inclusion of non-Brownian NMC particles does not significantly change the relaxation time of the aggregates. This is arguably supported by Figure 4, where both samples exhibit similar relaxation times.

In the reversible aggregation limit, *n* = 0.5, Zaccone et al. determined an equation for the critical attraction energy, $V_{c}$, between two colloidal particles required to have gelation at steady state. While the analysis above clearly indicates that the CB system is not within this limit, it is interesting to calculate a critical potential assuming the reversible aggregation limit is valid. $V_{c}$ is given by

$$-\frac{V_{c}}{k_{B}T}≅\ln\left[12{\left(\frac{\delta }{a}\right)}^{2}\phi_{0}\right]$$

where δ/a is the correlation length between voids normalized by the particle radius, and $\phi_{0}$ is the solid volume fraction. For CB and PVDF, we have estimated $V_{c}=+1\;k_{B}T$, from δ/a=1, $\delta =R_{g}=50$ nm and a=50 nm, and $\phi_{0}=0.02$ [28]. This result insinuates that the minimum interaction energy between particles is close to the diffusion energy, consistent with the observed flocculation and subsequent settling of CB in the absence of polymer. However, our experimental data suggests that we are between the two limits of reversible and irreversible aggregation, whereby the above equation for $V_{c}$ is not expected to hold.

**Figure 6:**
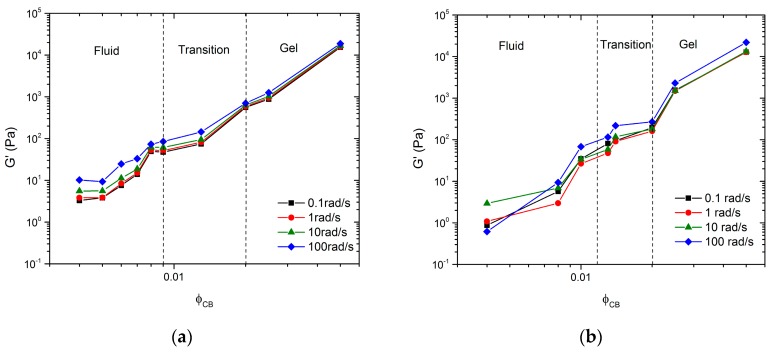
*G*’ vs. $\phi_{\mathrm{CB}}$ at 0.1, 1, 10, and 100 rad/s for (**a**) $\phi_{\mathrm{NMC}}=0$ and (**b**) $\phi_{\mathrm{NMC}}=0.26$.

Data from the frequency sweeps shown in Figure 2a,b can be plotted as the magnitude of *G*’ versus $\phi_{\mathrm{CB}}$ for specific frequencies. These representations for $\phi_{\mathrm{NMC}}=0$ and $\phi_{\mathrm{NMC}}=0.26$ are shown in Figure 6a,b, respectively. The figures identify three regimes: a fluid regime, a transition regime, and a strong gel regime. These regimes were identified using both frequency sweep data from Figure 2 and direct observations (see Section 3.1), similar to work done by Laurati et al. [24]. In Figure 6a, the fluid regime is defined as $\phi_{\mathrm{CB}}<0.009$ since these samples exhibit fluid-like behavior, denoted by the observation of particle sedimentation over time, by viscosity similar to the pure solvent, and low *G*’ and *G*” magnitudes. The transition regime is defined for $0.009\leq \phi_{\mathrm{CB}}<0.02$, similar to the observations of Laurati et al., since such samples showed a seemingly high viscosity compared to the pure solvent, but flowed when the vial was inverted [24]. The gel regime was defined for $\phi_{\mathrm{CB}}\geq 0.02$, since these samples exhibit characteristics of a strong gel; i.e., they do not flow when the sample vial is inverted and must be scooped instead of poured. Furthermore, *G*’ and *G*” are relatively flat and weak functions of ω, which is a typical indication of a network. In Figure 6b, for the mixed particle system, the fluid regime is defined as $\phi_{\mathrm{CB}}<0.013$ since all samples in this concentration regime showed fluid-like properties, i.e., a very little qualitative change in sample viscosity from the pure solvent. The transition regime is defined as $0.015\leq \phi_{\mathrm{CB}}<0.02$, since such samples exhibit much higher viscosities than expected for the given particle concentrations, but still flow when the vial is inverted. For $\phi_{\mathrm{CB}}\geq 0.02$, the samples again exhibit characteristics of a strong gel; i.e., they do not flow when inverted and show no signs of sedimentation. In both systems, we define $\phi_{gel}=0.02$ as the critical gelation limit. We used bright field microscopy images to better understand the microstructure of these regimes.

**Figure 7:**
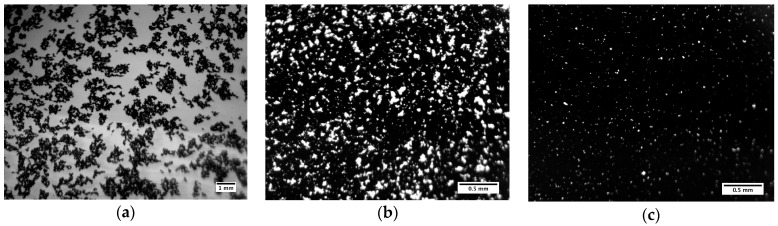
Bright field microscopy images for $\phi_{\mathrm{NMC}}=0$ samples (**a**) $\phi_{\mathrm{CB}}=0.005$; (**b**) $\phi_{\mathrm{CB}}=0.013$, and (**c**) $\phi_{\mathrm{CB}}=0.02$.

Figure 7 shows three bright field microscopy images for $\phi_{\mathrm{CB}}=$0.005, 0.013, and 0.02, representing the three regimes identified in rheology for the pure CB solutions. Attempts were made to take similar images of the mixed system; however, due to high NMC loading, little to no transmission of light was observed for all samples. It is evident from Figure 7a that $\phi_{\mathrm{CB}}=\;$0.005 does not show connectivity between carbon aggregates, while $\phi_{\mathrm{CB}}=\;$0.013 (Figure 7b) shows aggregate interconnectivity that appears to span the sample volume. Figure 7c shows a representative image of $\phi_{\mathrm{CB}}=$ 0.02, whereby it becomes very difficult to see any light penetrating the sample. At this volume fraction, the network is both volume spanning and dense. These qualitative observations confirm the different regimes depicted in Figure 6.

**Figure 8:**
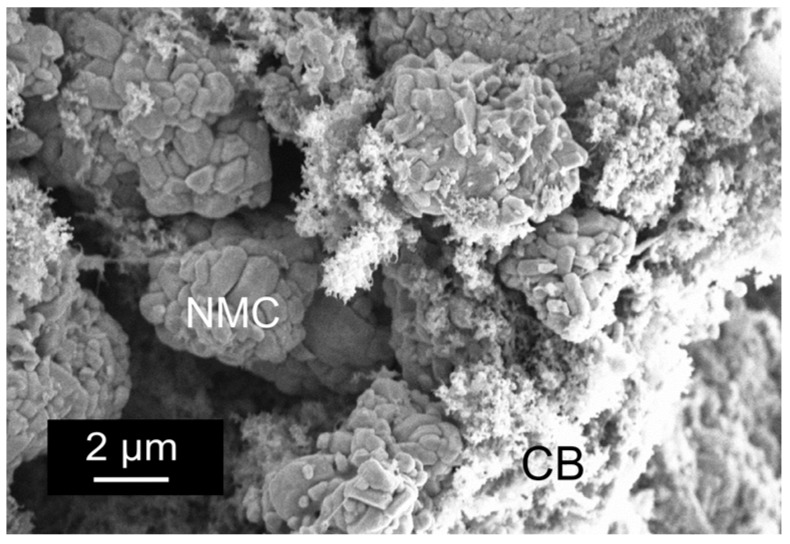
SEM image of the dried $\phi_{\mathrm{CB}}=0.025$ slurry, showing the coexistence of large NMC particles and the smaller CB aggregates. Note that the surface of the NMC is bare of CB aggregates.

The data clearly show that the inclusion of large non-Brownian particles at significantly high $\phi_{\mathrm{NMC}}$ causes little to no change in the three microstructure regimes. Furthermore, the inclusion of NMC has little to no effect on the mechanism of aggregation nor the critical time scale. The only noticeable change appears to be the lengthening of the fluid regime to higher $\phi_{\mathrm{CB}}$ and a compaction of the transition regime. Overall, the dependence of *G*’ on $\phi_{\mathrm{CB}}$ and the qualitative behavior of the samples appear the same. At first, this behavior seems counter-intuitive because it was hypothesized that the NMC particles would play an active role in the network formation. As a result, the carbon aggregates would only need to span the distance between particles of NMC instead of the entire solution volume, thus requiring fewer carbon particles to form a network and reducing $\phi_{\mathrm{gel}}$. Contrary to this hypothesis, Figure 8 shows a SEM image of a dried slurry with $\phi_{\mathrm{CB}}>\phi_{\mathrm{gel}}$. It is evident that the surface of the active material is clear of any noticeable aggregates of carbon black and that the carbon forms an independent network around the NMC particles. SEM therefore supports the rheology data that show that carbon particle network formation is unchanged with the inclusion of NMC particles.

Although no change in $\;\phi_{\mathrm{gel}}$ is observed, the inclusion of large NMC particles influences the shape of the LVE response and the amplitude sweep as seen in Figure 2 and Figure 3, respectively. The most drastic effect is seen in the stark differences between amplitude sweeps. Although the volume fractions are significantly below the glass transition, the gels formed by CB have tendencies that are similar to those of measurements performed on hard sphere glasses. In the case of $\phi_{\mathrm{NMC}}=0$, the shape of the amplitude sweep is like that of hard sphere glasses. In the case of $\phi_{\mathrm{NMC}}=0.26$, the shape of the amplitude sweep is like that of an attractive driven glass [25]. We hypothesize, similar to the arguments in Pham et al., that the low amplitude decrease of *G*’ for the mixed system at approximately γ= 1%  is caused by a rearrangement of the large NMC particles and that the high amplitude decrease is the rearrangement of the topology of nearest neighbors for CB [25]. Thus, the mixed colloidal systems allow for two-stage yielding, one for larger particles trapped in a network of smaller particles and the second for the smaller particle network.

An important problem in controlling suspension microstructure is predicting $\phi_{\mathrm{gel}}$, the particle volume fraction required to induce a phase change. In the work of Poon et al., $\phi_{\mathrm{gel}}{}^{*}$ can be predicted from Equation (1) assuming a strong interparticle attraction, which is supported by the analysis of Figure 5 [33]. Note that $\phi_{\mathrm{gel}}{}^{*}$ calculated from Equation (1) is essentially the crossover frequency when aggregation of particles occurs on a similar timescale to settling due to gravity (a necessary constraint if a volume spanning network is to form). We therefore expect $\phi_{\mathrm{gel}}{}^{*}$ to be closer to our transition regime than to our gelation regime. In all samples, *c*_p_ = 48 mg/mL, while *c*_p_*, the overlap concentration, was calculated to be 0.8 mg/mL [23,28]. Thus, for all samples studied here, Equation (1) is expected to be applicable. Equation (1) shows that $\phi_{\mathrm{gel}}$ is extremely sensitive to particle size *a*, but industrial battery materials such as CB are polydisperse and nonideal. Based on the TEM in Figure 1a, small (*a* < 20 nm) primary particles of CB form secondary particles of 40 < *a* < 200 nm. Some of these secondary particles may consist of primary particles that are chemically fused during synthesis [41]. The secondary particles form the larger aggregates pictured in Figure 1a. These larger aggregates are expected to break up when sonicated, resulting in the DLS-measured diameter of 100 nm. The particle radius that governs the rheological response of the slurry is therefore not immediately apparent.

Predicting $\phi_{\mathrm{gel}}$ also requires knowledge of the fractal dimension *D*_f_. In the case of diffusion-limited cluster aggregation (irreversible aggregation mechanism *n* = 0), *D*_f_ is expected to be between 1.7 and 1.9 [33]. *D*_f_ is typically determined from X-ray, neutron, or light scattering data using $I\sim q^{-D_{f}}$, where *I* is the measured intensity, and *q* is the scattering vector [27]. Unfortunately, the CB particle size is too big to measure on our current SAXS setup, as the available *q* range only probes the particle diameter dimensions (~100 nm). Light scattering is a possibility, but light transmission through the sample requires a special sample holder with a smaller path length (and is currently under investigation). An alternative method of obtaining *D*_f_ is optical microscopy image analysis [27]. Box-counting analysis [42] of Figure 7a yields 1.7 < *D*_f_ < 1.8. If *D*_f_ < 2, a two-dimensional projection should yield the same *D*_f_ as the three-dimensional structure [27].

**Figure 9:**
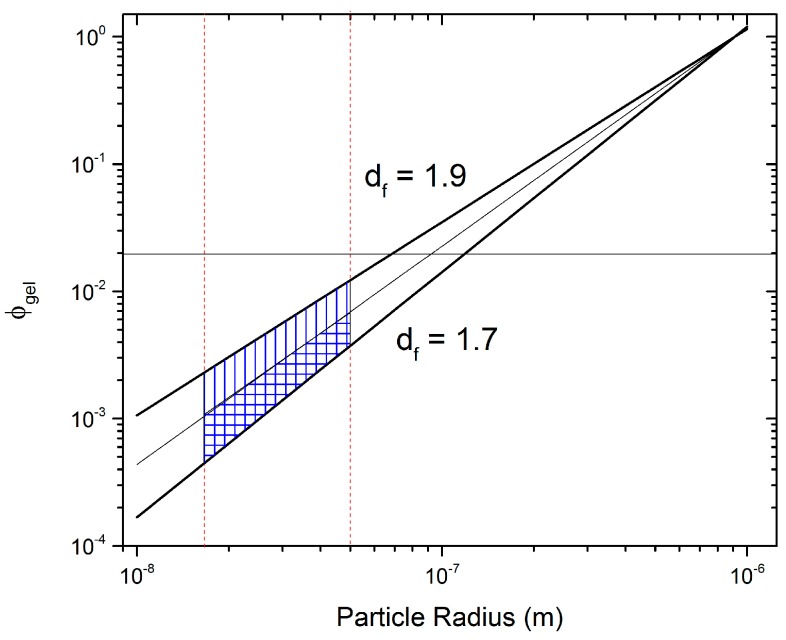
Predicted critical gelation concentration from Equation (1) considering 1.7 < *D*_f_ < 1.9 (black sloped lines) and CB particle radius of 15 to 50 nm (vertical red dashed lines). The shaded region is the predicted gelation regime. The horizontal black line is the experimentally measured $\phi_{\mathrm{gel}}$.

To account for uncertainties in *a* and *D*_f_, $\phi_{\mathrm{gel}}$ was predicted using Equation (1) for 15 nm < *a* < 150 nm (vertical red lines in Figure 9) and 1.7 < *D*_f_ < 1.9 (sloped black lines in Figure 9). Figure 9 clearly shows a shaded region that depicts the range of predictions for Equation (1) for the specified parameters. Using the DLCA range for *D*_f_ and an intermediate *a* = 50 nm, a range for the potential $\phi_{\mathrm{gel}}$ of CV can be predicted to lie between 0.0004 and 0.01. The upper limit is surprisingly close to the result obtained from rheology, showing a transition regime at $\phi_{\mathrm{gel}}>0.01$, where essentially aggregation is very close to the settling time of particles due to gravity, but not yet enough to create a volume spanning network. Note that the agreement between Equation (1) and the experimentally observed transition regime is only similar when the upper limit is considered. We hypothesize that while the primary CB particle size is on average less than 50 nm in radius, the secondary particles are in fact the dominant species forming aggregates and are much larger than 50 nm, which could explain the agreement with the upper limit.

In conclusion, our rheological measurements and analysis show that the aggregation mechanism, critical volume fraction, and aggregate relaxation time of CB in NMP are not strongly influenced by the presence of high volume fractions of non-Brownian NMC particles. SEM indicates that this is because the large particles do not participate in the percolating network. The presence of NMC particles influences the shape of both the linear viscoelastic and nonlinear response of the materials. Comparison of our data with the theoretical prediction of Poon et al. show excellent agreement for the CB-only case. Our finding has beneficial implications for battery formulation, among other fields, because it suggests that the microstructure of the electrode slurry can be controlled by the CB concentration, independent of the active material. Efforts in our lab to tune slurry and electrode microstructures by manipulating $\phi_{\mathrm{CB}}$ and their relation to battery performance are ongoing.
